# Supplementary figures and images for: The PilT retraction ATPase promotes both extension and retraction of the MSHA type IVa pilus in Vibrio cholerae
Source: PLoS Genet. 2022 Dec 21;18(12):e1010561. doi: 10.1371/journal.pgen.1010561 (PMC9815625; doi:10.1371/journal.pgen.1010561)

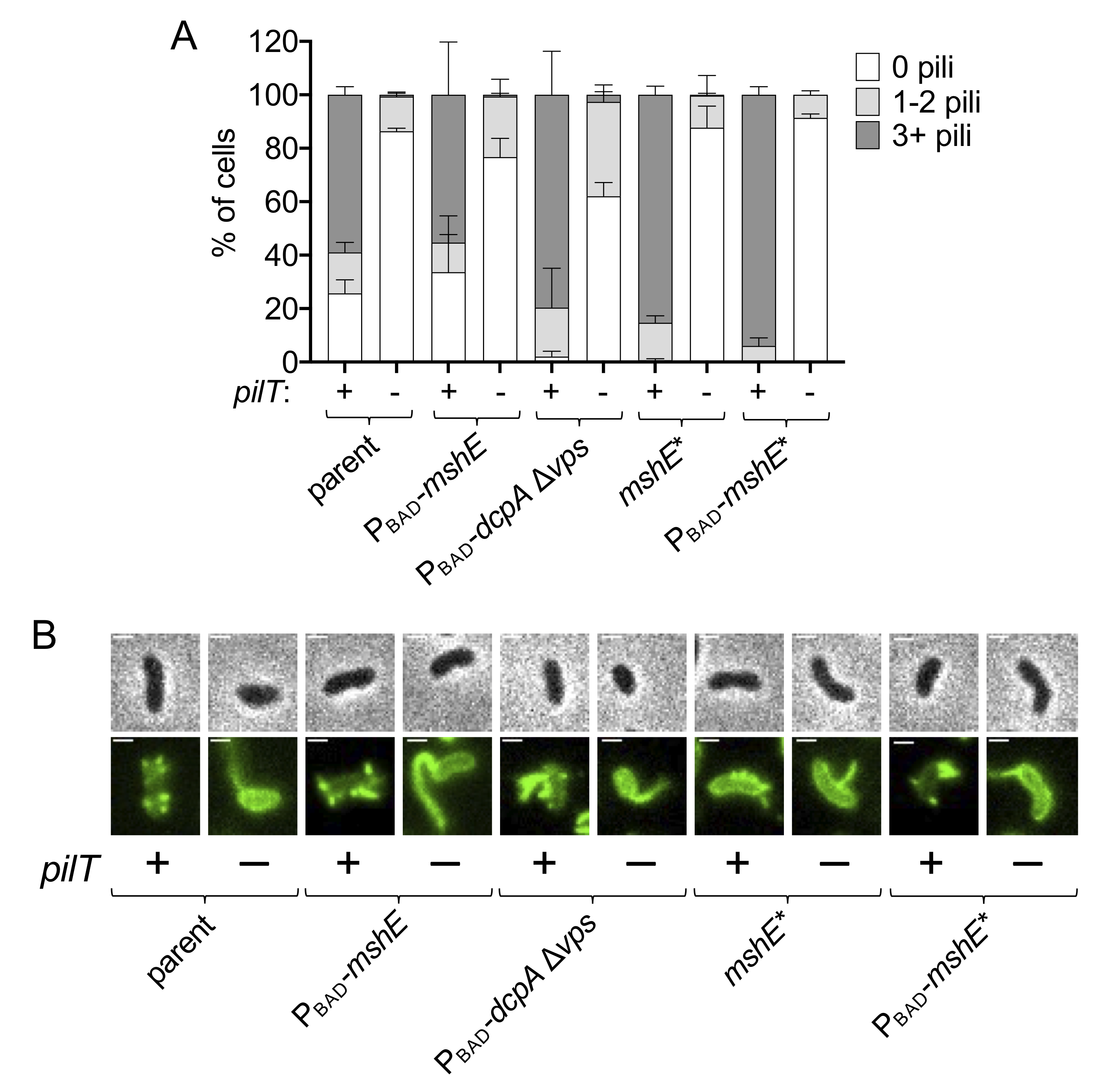

Supplement: S1 Fig — (A) Quantification of piliation in strains with altered extension motor regulation. Strains that retain native pilT are denoted “+” and strains with ΔpilT mutations are denoted “-”. Cells were categorized as either having no pili (white bars), 1–2 pili (light gray bars), or at least 3 pili (dark gray bars). n = 300 cells analyzed from three independent biological replicates for all samples and data are displayed as the mean ± SD. All strains with ectopic PBAD constructs were induced with 0.2% arabinose. Parent and ΔpilT data from Fig 1C are included for comparison. (B) Representative images of piliated cells from strains in A. Phase images (top) show cell boundaries and fluorescence images (bottom) show AF488-mal labeled pili. Strains that retain native pilT are denoted “+” and strains with ΔpilT mutations are denoted “-”. Scale bar = 1 μm. (TIFF) [file pgen.1010561.s001.tiff]

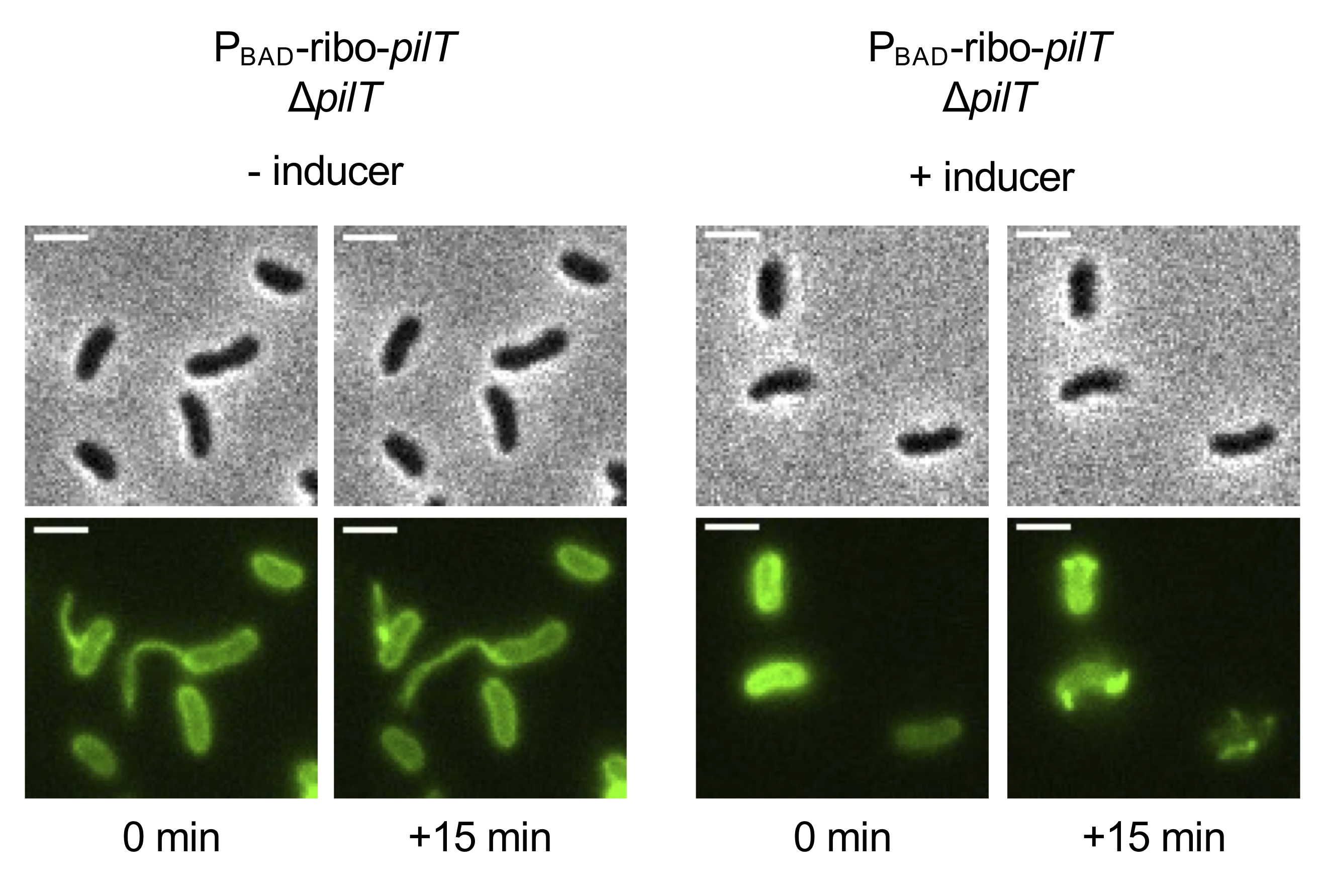

Supplement: S2 Fig — Representative images of a PBAD-ribo-pilT ΔpilT strain when pilT expression is rapidly induced under the microscope. Phase images (top) show cell boundaries and fluorescence images (bottom) show AF488-mal labeled pili. Samples were applied to a slide with a gelzan pad either lacking inducer (left; “- inducer”) or including 0.2% arabinose and 1.5mM theophylline as an inducer (“+ inducer”). Each sample was imaged immediately after application of the gelzan pad (“0 min”) and again after 15 min (“+15 min”). Scale bar = 2 μm. (TIFF) [file pgen.1010561.s002.tiff]

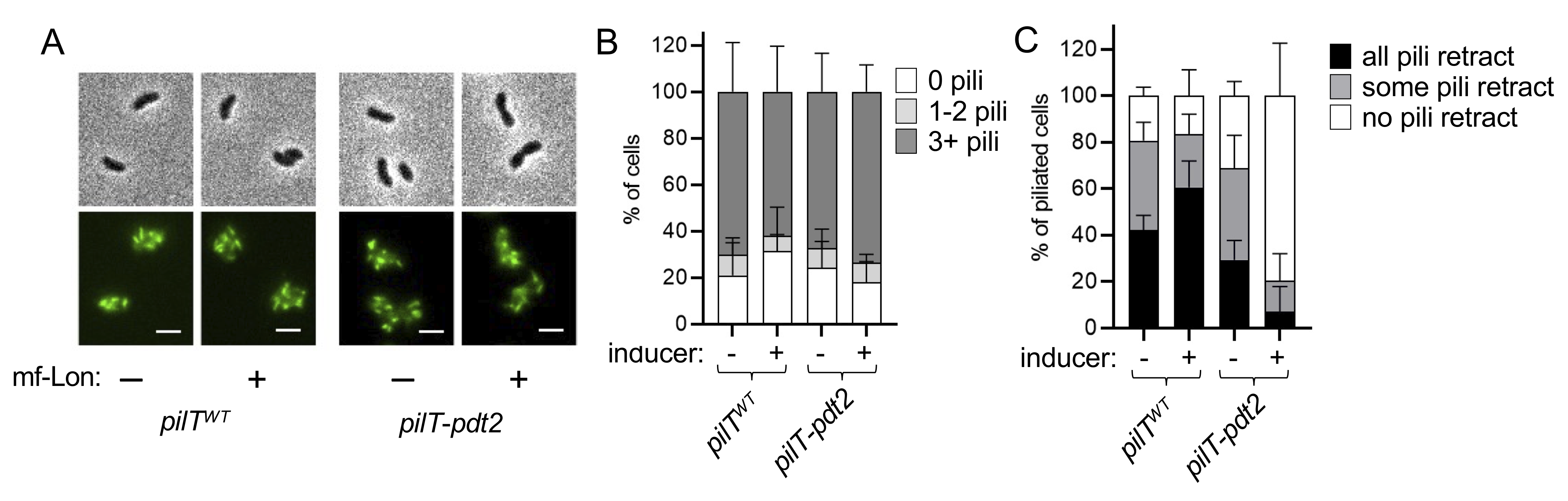

Supplement: S3 Fig — (A) Representative images of cells where mf-Lon was (“+”; 0.2% arabinose) or was not (“-”) induced. Phase images (top) show cell boundaries and fluorescence images (bottom) show AF488-mal labeled pili. Scale bar = 1 μm. (B) Quantification of piliation in samples from A. Cells were categorized as either having no pili (white bars), 1–2 pili (light gray bars), or at least 3 pili (dark gray bars). n = 300 cells analyzed from three independent biological replicates for all samples. (C) Quantification of imaging-induced retraction in samples from A. Graph displays the percentage of cells in each replicate that retracted all, some, or no pili during a three-minute timelapse. n ≥ 25 piliated cells analyzed for each of the three biological replicates. All data are displayed as the mean ± SD. (TIFF) [file pgen.1010561.s003.tiff]

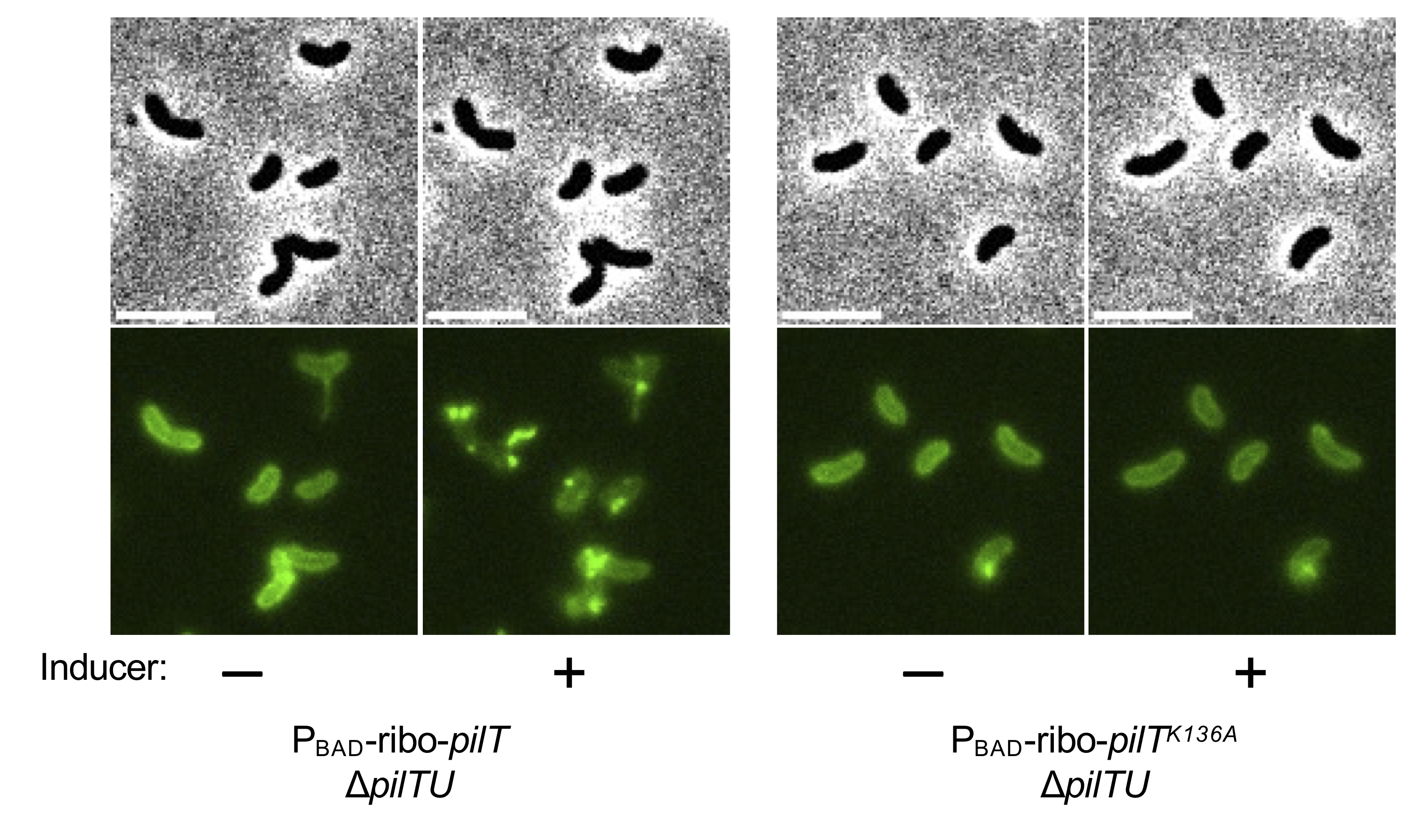

Supplement: S4 Fig — Fluorescence microscopy of the indicated strains. Phase images (top) show cell boundaries and fluorescence images (bottom) show AF488-mal labeled pili. Each sample is shown before (-) and after (+) induction. Scale bar = 4 μm. Data are representative of three independent experiments. (TIFF) [file pgen.1010561.s004.tiff]

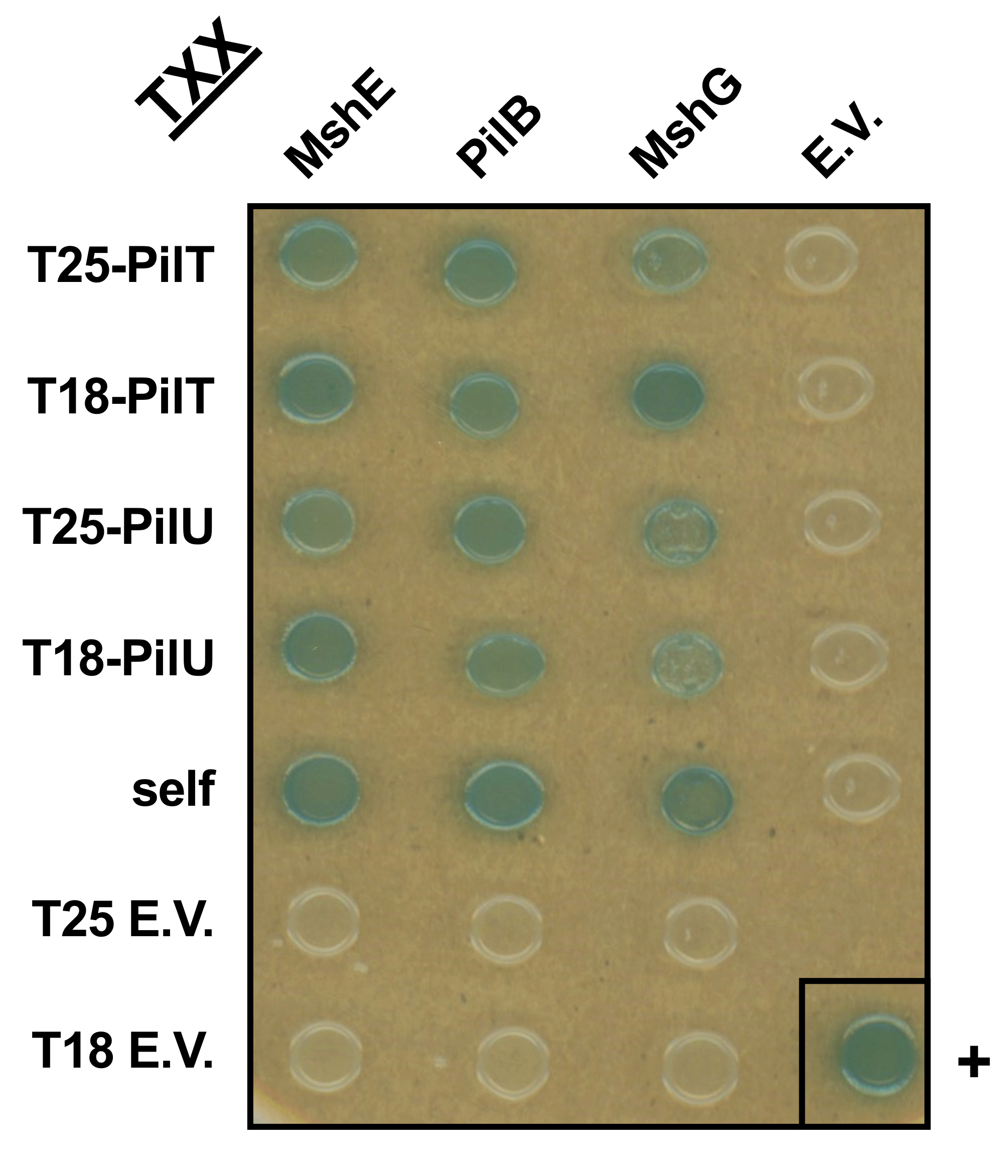

Supplement: S5 Fig — Representative image of a BACTH assay between T25- and T18- fusions of the indicated proteins. Both PilT and PilU displayed strong interactions with themselves (“self”), the extension motors (MshE and PilB), as well as the MSHA platform protein (MshG). “E.V.” denotes a pairing with an empty vector, and “+” indicates the BACTH positive control (T25-Zip + T18-Zip). This image is representative of three independent experiments. (TIFF) [file pgen.1010561.s005.tiff]

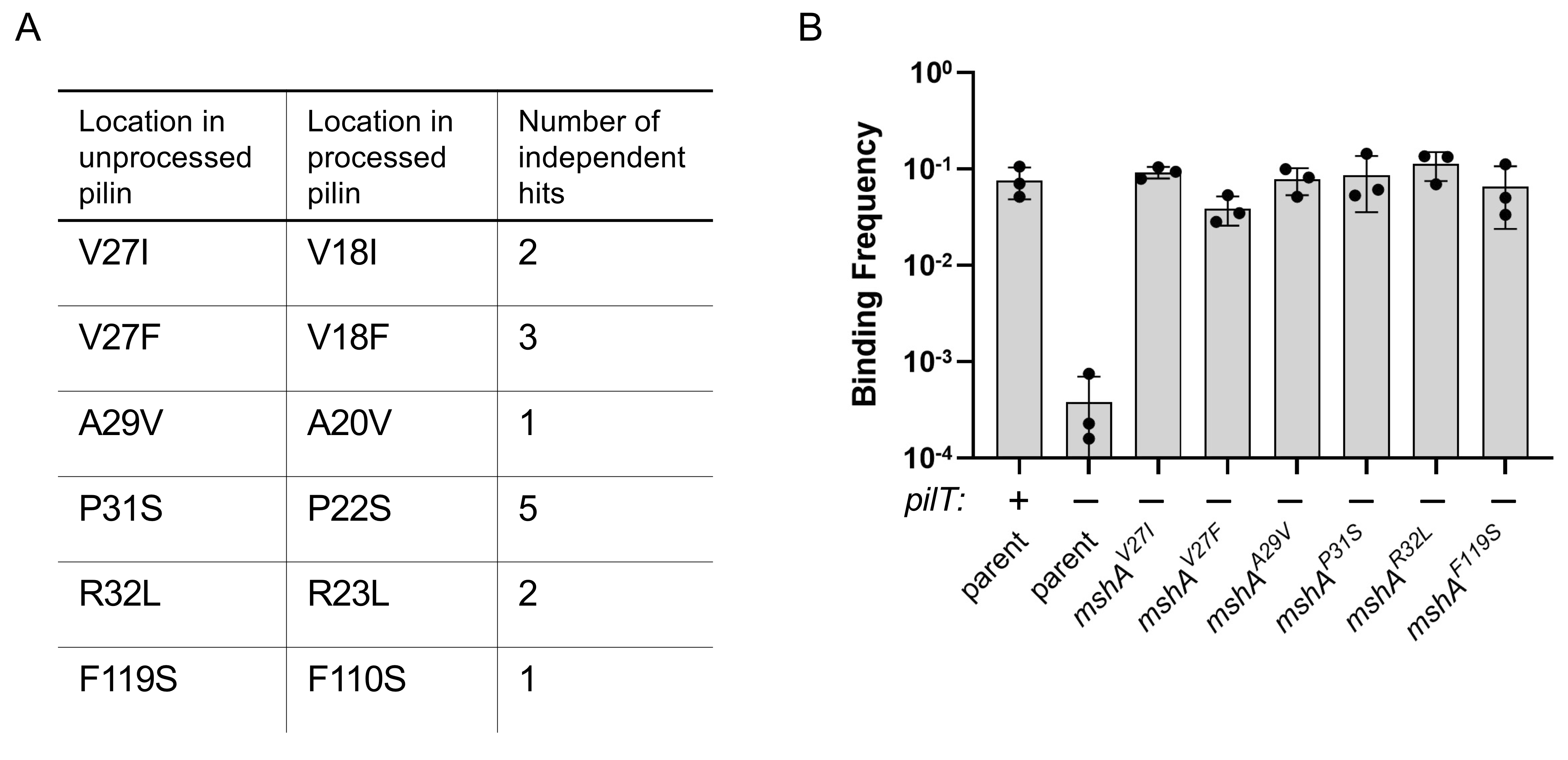

Supplement: S6 Fig — (A) Table of the MshA mutations isolated in the ΔpilT suppressor screen. The relative position of the mutated residues is indicated relative to the MshA start codon (location in unprocessed pilin) and relative to the first amino acid in the pilin after being processed by the pre-pilin peptidase (location in processed pilin).”Number of independent hits” denotes the number of distinct genetic lines in which the indicated suppressor mutation was isolated. (B) Binding frequency of the indicated strains to the wall of culture tubes. Strains that retain native pilT are denoted “+” and strains with ΔpilT mutations are denoted “-”. Data are from three independent biological replicates and shown as the mean ± SD. (TIFF) [file pgen.1010561.s006.tiff]

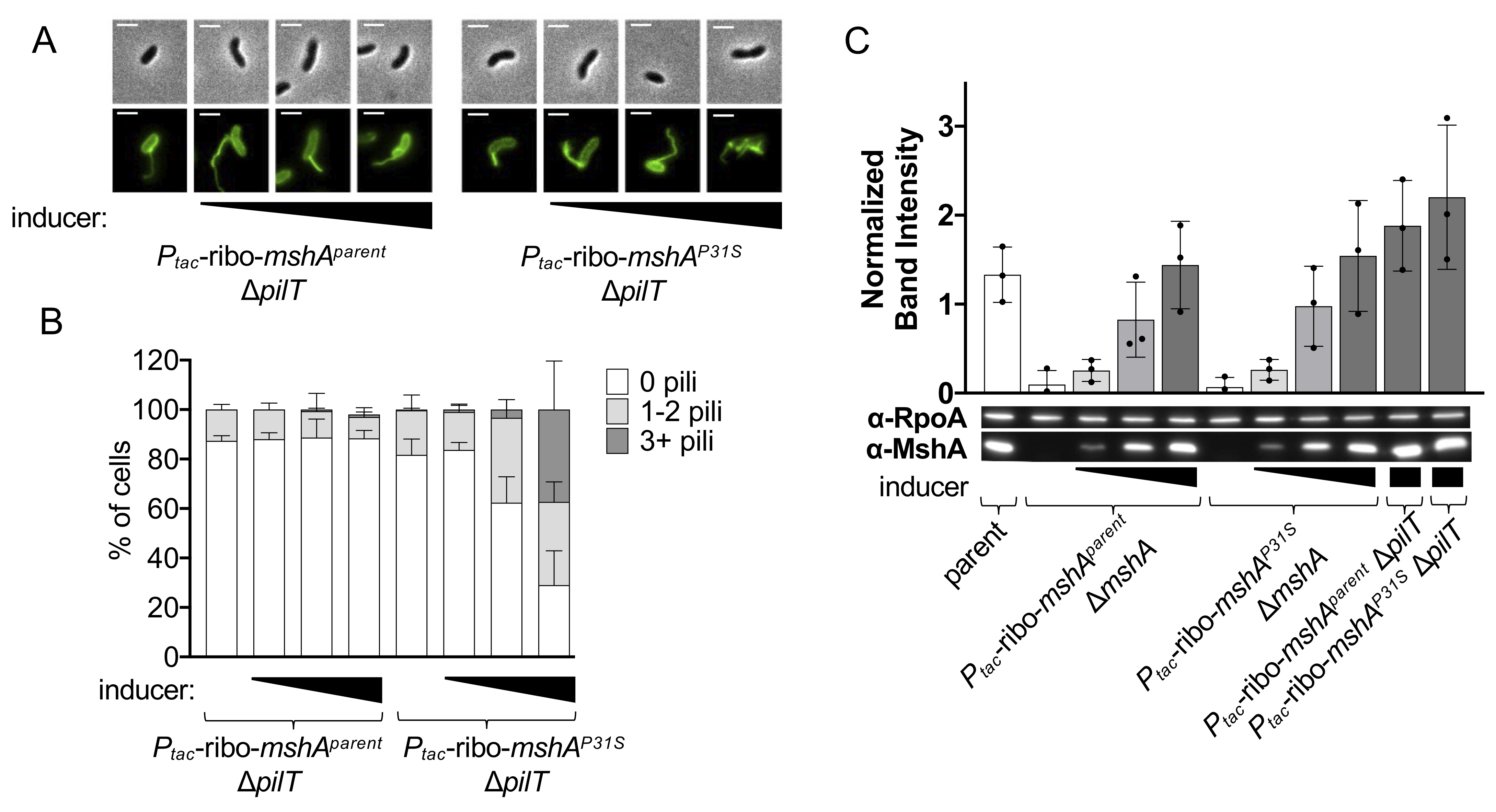

Supplement: S7 Fig — (A) Representative images of piliated cells from strains under increasing induction of either Ptac-riboswitch-mshAparent or Ptac-riboswitch-mshAP31S. All mshA alleles in these strains (native and ectopic) contain the T70C mutation needed for AF488-mal labeling. Concentrations of inducer used from left to right: (1) 0 μM IPTG + 0 μM theophylline, (2) 5 μM IPTG + 75 μM theophylline, (3) 20 μM IPTG + 300 μM theophylline, and (4) 100 μM IPTG + 1.5 mM theophylline. Phase images (top) show cell boundaries and fluorescence images (bottom) show AF488-mal labeled pili. Scale bar = 2 μm. (B) Quantification of piliation in samples from A. Cells were categorized as either having no pili (white bars), 1–2 pili (light gray bars), or at least 3 pili (dark gray bars). n = 300 cells analyzed from three independent biological replicates for all samples. (C) Western blot quantification of cell-associated MshA in the indicated strains in the induction conditions used in A and B. Band intensities are normalized to the RpoA loading control. Data are from three independent biological replicates. All data are displayed as the mean ± SD. (TIFF) [file pgen.1010561.s007.tiff]

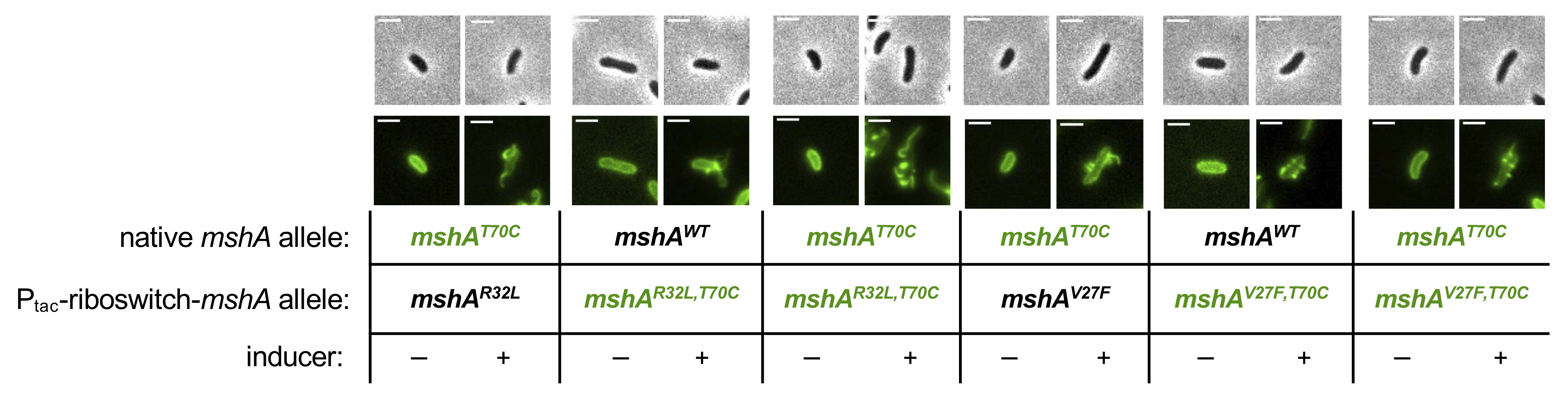

Supplement: S8 Fig — Representative images of cells from ΔpilT strains expressing the indicated mshA allele at the native locus (native mshA allele) and ectopic locus (Ptac-riboswitch-mshA allele). Cells were either grown with (”+”) or without (”-”) 100 μM IPTG + 1.5 mM theophylline to induce the ectopic Ptac-riboswitch-mshA allele in the strain as indicated. Only the alleles containing the T70C mutation can be labeled with AF488-mal and are denoted in green text in the table below the images. Scale bar = 2 μm. (TIFF) [file pgen.1010561.s008.tiff]
