# Supplementary material for: The PilT retraction ATPase promotes both extension and retraction of the MSHA type IVa pilus in Vibrio cholerae
Source: PLoS Genet. 2022 Dec 21;18(12):e1010561. doi: 10.1371/journal.pgen.1010561 (PMC9815625; doi:10.1371/journal.pgen.1010561)
Supplement: S1 Table — (PDF) [file pgen.1010561.s009.pdf]

S1 Table. Strains used in this study

Strain name in manuscript	Figure	Genotype and antibiotic resistances	Strain #	Reference
parent (MSHA T4aP)	Fig. 1A, 1B, 1C, 2A, 2B, 3A, 3B, 4A, 4B, 4C, 6B, 6C, 6D, S1A, S1B, S6B, S7C	E7946 Sm^R, ΔVC1807::Kan^R, *mshA*^{T70C}	CAH764 (SAD3125)	[1,2]
Δ*pilT* (MSHA T4aP)	Fig. 1A, 1B, 1C, 2A, 2B, 3A, 3B, 4A, 4B, 4C, 6B, 6C, 6D, S1A, S1B, S6B	E7946 Sm^R, ΔVC1807::Kan^R, *mshA*^{T70C}, Δ*pilT*::Tm^R	HKQ001 (SAD3144)	[1,2,3]
Parent (competence T4aP)	Fig. 1A, 1B, 1C	E7946 Sm^R, ΔVC1807::Zeo^R, Δ*lacZ*::*lacIq*, P_{tac}-*tfoX*, Δ*luxO*::miniFRT, *pilA*^{S56C}	TND0905 (SAD2468)	[3]
Δ*pilT* (competence T4aP)	Fig. 1A, 1B, 1C	E7946 Sm^R, ΔVC1807::Zeo^R, Δ*lacZ*::*lacIq*, P_{tac}-*tfoX*, Δ*luxO*::miniFRT, *pilA*^{S56C}, ΔpilT::Tm^R	TND1035 (SAD2469)	[3]
Δ*mshE*	Fig. 2B	E7946 Sm^R, ΔVC1807::Kan^R, *mshA*^{T70C}, Δ*mshE*::miniFRT	TND0503 (SAD3198)	[2]
unlabelable	Fig. 2B	E7946 Sm^R	SAD030	[4]
P_{BAD}-*mshE* Δ*mshE*	Fig. 2C, 2D, S1A, S1B	E7946 Sm^R, ΔVC1807::Cm^R, *mshA*^{T70C}, Δ*mshE*::miniFRT, Δ*lacZ*::P_{BAD}-*mshE* $Carb^R$	HKQ196 (SAD3321)	This study
P_{BAD}-*mshE* Δ*mshE* Δ*pilT*	Fig. 2C, 2D, S1A, S1B	E7946 Sm^R, ΔVC1807::Cm^R, *mshA*^{T70C}, Δ*mshE*::miniFRT, Δ*lacZ*::P_{BAD}-*mshE* $Carb^R$, Δ*pilT*::Tm^R	HKQ496 (SAD3322)	This study
P_{BAD}-ribo-*pilT*	Fig. 3A, 3B	E7946 Sm^R, ΔVC1807::Kan^R, *mshA*^{T70C}, ΔVCA0692::P_{BAD}-riboswitch-*pilT* $Carb^R$	HKQ483 (SAD3303)	This study
P_{BAD}-ribo-*pilT* Δ*pilT*	Fig. 3A, 3B, S2	E7946 Sm^R, ΔVC1807::Kan^R, *mshA*^{T70C}, ΔVCA0692::P_{BAD}-riboswitch-*pilT* $Carb^R$, ΔpilT::Tm^R	HKQ484 (SAD3304)	This study
mshJ-mCherry	Fig. 3C	E7946 Sm^R, ΔVC1807::$Spec^R$, *mshA*^{T70C}, *mshJ-mCherry*	TND3462 (SAD3328)	This study
mshJ-mCherry Δ*pilT*	Fig. 3C	E7946 Sm^R, ΔVC1807::$Spec^R$, *mshA*^{T70C}, *mshJ-mCherry*, Δ*pilT*::Tm^R	TND3500 (SAD3329)	This study
Δ*pilU*	Fig. 4A, 4B, 4C	E7946 Sm^R, ΔVC1807::Cm^R, *mshA*^{T70C}, Δ*pilU*::miniFRT	TND0471 (SAD3305)	This study
Δ*pilTU*	Fig. 4A, 4B, 4C	E7946 Sm^R, ΔVC1807::Kan^R, *mshA*^{T70C}, Δ*pilTU*::Tm^R	JLC419 (SAD3306)	This study
pilT^{K136A}	Fig. 4A, 4B, 4C	E7946 Sm^R, ΔVC1807::$Spec^R$, *mshA*^{T70C}, *pilT*^{K136A}	HKQ006 (SAD3307)	This study
pilT^{K136A} Δ*pilU*	Fig. 4A, 4B, 4C	E7946 Sm^R, ΔVC1807::Kan^R, *mshA*^{T70C}, *pilT*^{K136A}, Δ*pilU*::Tm^R	JLC446 (SAD3308)	This study
pilU^{K134A}	Fig. 4A, 4B, 4C	E7946 Sm^R, ΔVC1807::$Spec^R$, *mshA*^{T70C}, *pilU*^{K134A}	TND1093 (SAD3411)	This study
pilT^{K136A} *pilU*^{K134A}	Fig. 4A, 4B, 4C	E7946 Sm^R, ΔVC1807::$Spec^R$, *mshA*^{T70C}, *pilT*^{K136A}, *pilU*^{K134A}	TND1095 (SAD3412)	This study
parent	Fig. 5A	A1552, ΔVC1807::Zeo^R, *mshA*^{T70C}, Δ*mshE*::miniFRT, Δ*lacZ*::P_{BAD}-*mshE*$^{L10/54/58A}$ Cm^R, Δ*pilU*::Tm^R	HKQ535 (SAD3413)	This study
pilT ^{slow}	Fig. 5A	A1552, ΔVC1807::Zeo^R, *mshA*^{T70C}, Δ*mshE*::miniFRT, Δ*lacZ*::P_{BAD}-*mshE*$^{L10/54/58A}$ Cm^R, *pilT*^{L201C}, Δ*pilU*::Tm^R	HKQ537 (SAD3414)	This study
mshE ^{slow}	Fig. 5A	A1552, ΔVC1807::Zeo^R, *mshA*^{T70C}, Δ*mshE*::miniFRT, Δ*lacZ*::P_{BAD}-*mshE*$^{L10/54/58A, L390C}$ Cm^R, Δ*pilU*::Tm^R	HKQ538 (SAD3415)	This study

pilT ^{slow} *mshE* ^{slow}	Fig. 5A	A1552, ΔVC1807::Zeo^{R}, *mshA*^{T70C}, Δ*mshE*::miniFRT, Δ*lacZ*::P$_{BAD}$-*mshE*$^{L10/54/58A, L390C}$ Cm^{R}, *pilT*^{L201C}, Δ*pilU*::Tm^{R}	HKQ539 (SAD3416)	This study
Δ*mshE*, pLTetO-*mshE*, *pilT*-pdt2, P$_{BAD}$-*mf-Lon*	Fig. 5B, 5C, 5D, 5E	E7946 Sm^{R}, ΔVC1807::Kan^{R}, *mshA*^{T70C}, Δ*mshE*::miniFRT, Δ*lacZ*::pLTetO-*mshE* Spec^{R}, ΔVCA0692::P$_{BAD}$-*mf-lon* Carb^{R}, *pilT*-pdt2	TND3675 (SAD3417)	This study
mshA^{V27I}	Fig. 6B, 6C, 6D	E7946 Sm^{R}, ΔVC1807::Spec^{R}, *mshA*$^{T70C, V27I}$	HKQ025 (SAD3309)	This study
mshA^{V27I} Δ*pilT*	Fig. 6B, 6C, 6D, S6B	E7946 Sm^{R}, ΔVC1807::Spec^{R}, *mshA*$^{T70C, V27I}$, Δ*pilT*::Tm^{R}	HKQ031 (SAD3310)	This study
mshA^{V27F}	Fig. 6B, 6C, 6D	E7946 Sm^{R}, ΔVC1807::Spec^{R}, *mshA*$^{T70C, V27F}$	HKQ026 (SAD3311)	This study
mshA^{V27F} Δ*pilT*	Fig. 6B, 6C, 6D, S6B	E7946 Sm^{R}, ΔVC1807::Spec^{R}, *mshA*$^{T70C, V27F}$, Δ*pilT*::Tm^{R}	HKQ032 (SAD3312)	This study
mshA^{A29V}	Fig. 6B, 6C, 6D	E7946 Sm^{R}, ΔVC1807::Spec^{R}, *mshA*$^{T70C, A29V}$	HKQ027 (SAD3313)	This study
mshA^{A29V} Δ*pilT*	Fig. 6B, 6C, 6D, S6B	E7946 Sm^{R}, ΔVC1807::Spec^{R}, *mshA*$^{T70C, A29V}$, Δ*pilT*::Tm^{R}	HKQ033 (SAD3314)	This study
mshA^{P31S}	Fig. 6B, 6C, 6D	E7946 Sm^{R}, ΔVC1807::Spec^{R}, *mshA*$^{T70C, P31S}$	HKQ028 (SAD3315)	This study
mshA^{P31S} Δ*pilT*	Fig. 6B, 6C, 6D, S6B	E7946 Sm^{R}, ΔVC1807::Spec^{R}, *mshA*$^{T70C, P31S}$, Δ*pilT*::Tm^{R}	HKQ034 (SAD3316)	This study
mshA^{R32L}	Fig. 6B, 6C, 6D	E7946 Sm^{R}, ΔVC1807::Spec^{R}, *mshA*$^{T70C, R32L}$	HKQ029 (SAD3317)	This study
mshA^{R32L} Δ*pilT*	Fig. 6B, 6C, 6D, S6B	E7946 Sm^{R}, ΔVC1807::Spec^{R}, *mshA*$^{T70C, R32L}$, Δ*pilT*::Tm^{R}	HKQ035 (SAD3318)	This study
mshA^{F119S}	Fig. 6B, 6C, 6D	E7946 Sm^{R}, ΔVC1807::Spec^{R}, *mshA*$^{T70C, F119S}$	HKQ030 (SAD3319)	This study
mshA^{F119S} Δ*pilT*	Fig. 6B, 6C, 6D, S6B	E7946 Sm^{R}, ΔVC1807::Spec^{R}, *mshA*$^{T70C, F119S}$, Δ*pilT*::Tm^{R}	HKQ036 (SAD3320)	This study
mshA^{T70C} P$_{tac}$-ribo-*mshA*^{WT} Δ*pilT*	Fig. 7	E7946 Sm^{R}, ΔVC1807::Spec^{R}, *mshA*^{T70C}, Δ*lacZ*::P$_{tac}$-riboswitch-*mshA*^{C70T} Kan^{R}, Δ*pilT*::Tm^{R}	HKQ531 (SAD3338)	This study
mshA^{WT} P$_{tac}$-ribo-*mshA*^{T70C} Δ*pilT*	Fig. 7	E7946 Sm^{R}, Δ*lacZ*::P$_{tac}$-riboswitch-*mshA*^{T70C} Kan^{R}, Δ*pilT*::Tm^{R}	HKQ529 (SAD3339)	This study
mshA^{T70C} P$_{tac}$-ribo-*mshA*^{P31S} Δ*pilT*	Fig. 7	E7946 Sm^{R}, *mshA*^{T70C}, ΔVC1807::Kan^{R}, Δ*lacZ*::P$_{tac}$-riboswitch-*mshA*$^{P31S, C70T}$ Cm^{R}, Δ*pilT*::Tm^{R}	HKQ526 (SAD3340)	This study
mshA^{WT} P$_{tac}$-ribo-*mshA*$^{P31S, T70C}$ Δ*pilT*	Fig. 7	E7946 Sm^{R}, Δ*lacZ*::P$_{tac}$-riboswitch-*mshA*$^{P31S, T70C}$ Kan^{R}, Δ*pilT*::Tm^{R}	HKQ533 (SAD3341)	This study
P$_{tac}$-ribo-*mshA*^{P31S} Δ*pilT*	Fig. 7, S7A, S7B, S7C	E7946 Sm^{R}, ΔVC1807::Spec^{R}, *mshA*^{T70C}, Δ*lacZ*::P$_{tac}$-riboswitch-*mshA*$^{T70C, P31S}$ Kan^{R}, Δ*pilT*::Tm^{R}	HKQ106 (SAD3335)	This study
P$_{BAD}$-*dcpA* Δ*vps*	Fig. S1A, S1B	E7946 Sm^{R}, ΔVC1807::Kan^{R}, *mshA*^{T70C}, Δ*vps-rbmA*::Zeo^{R}, ΔVCA0692::P$_{BAD}$-*dcpA* Carb^{R}	TND3247 (SAD3323)	This study
P$_{BAD}$-*dcpA* Δ*vps* Δ*pilT*	Fig. S1A, S1B	E7946 Sm^{R}, ΔVC1807::Kan^{R}, *mshA*^{T70C}, Δ*vps-rbmA*::Zeo^{R}, ΔVCA0692::P$_{BAD}$-*dcpA* Carb^{R}, Δ*pilT*::Tm^{R}	HKQ494 (SAD3324)	This study
*mshE**	Fig. S1A, S1B	E7946 Sm^{R}, ΔVC1807::Cm^{R}, *mshA*^{T70C}, *mshE*$^{L10/54/58A}$	SAD1756	[2]
*mshE** Δ*pilT*	Fig. S1A, S1B	E7946 Sm^{R}, ΔVC1807::Cm^{R}, *mshA*^{T70C}, *mshE*$^{L10/54/58A}$, Δ*pilT*::Tm^{R}	HKQ495 (SAD3325)	This study
P$_{BAD}$-*mshE**	Fig. S1A, S1B	E7946 Sm^{R}, ΔVC1807::Cm^{R}, *mshA*^{T70C}, Δ*lacZ*::P$_{BAD}$-*mshE*$^{L10/54/58A}$ Carb^{R}, Δ*mshE*::miniFRT	HKQ181 (SAD3326)	This study
P$_{BAD}$-*mshE** Δ*pilT*	Fig. S1A, S1B	E7946 Sm^{R}, ΔVC1807::Cm^{R}, *mshA*^{T70C}, Δ*lacZ*::P$_{BAD}$-*mshE*$^{L10/54/58A}$ Carb^{R}, Δ*mshE*::miniFRT, Δ*pilT*::Tm^{R}	HKQ497 (SAD3327)	This study

pilT^{WT}	Fig. S3A, S3B, S3C	E7946 Sm^{R}, ΔVC1807::Kan^{R}, *mshA*^{T70C}, ΔVCA0692::P$_{BAD}$-*mf-lon* CarbR	HKQ367 (SAD3330)	This study
pilT-pdt2	Fig. S3A, S3B, S3C, S3D	E7946 Sm^{R}, ΔVC1807::Kan^{R}, *mshA*^{T70C}, ΔVCA0692::P$_{BAD}$-*mf-lon* CarbR, *pilT*-pdt2	HKQ368 (SAD3331)	This study
P$_{BAD}$-ribo-*pilT* Δ*pilTU*	Fig. S4	E7946 Sm^{R}, ΔVC1807::Kan^{R}, *mshA*^{T70C}, ΔVCA0692::P$_{BAD}$-riboswitch-*pilT* Carb^{R}, Δ*pilTU*::Tm^{R}	TND3617 (SAD3418)	This study
P$_{BAD}$-ribo-*pilT*^{K136A} Δ*pilTU*	Fig. S4	E7946 Sm^{R}, ΔVC1807::Kan^{R}, *mshA*^{T70C}, ΔVCA0692::P$_{BAD}$-riboswitch-*pilT*^{K136A} Carb^{R}, Δ*pilTU*::Tm^{R}	TND3641 (SAD3419)	This study
T25-PilT	Fig. S5	TG1, pKT25-PilT Kan^{R}	SAD2248	[3]
T18-PilT	Fig. S5	TG1, pUT18C-PilT Carb^{R}	SAD2249	[3]
T25-PilU	Fig. S5	TG1, pKT25-PilU Kan^{R}	SAD2250	[3]
T18-PilU	Fig. S5	TG1, pUT18C-PilU Carb^{R}	SAD2251	[3]
T25 E.V.	Fig. S5	TG1, pKT25 vector Kan^{R}	SAD2236	[3]
T18 E.V.	Fig. S5	TG1, pUT18C vector Carb^{R}	SAD2240	[3]
MshE-T25	Fig. S5	TG1, MshE-pKT25 Kan^{R}	HKQ513 (SAD3332)	This study
MshE-T18	Fig. S5	TG1, MshE-pUT18C Carb^{R}	HKQ515 (SAD3333)	This study
T25-PilB	Fig. S5	TG1, pKT25-PilB Kan^{R}	EHC052 (SAD3264)	[5]
T18-PilB	Fig. S5	TG1, pUT18C-PilB Carb^{R}	EHC050 (SAD3265)	[5]
T25-MshG	Fig. S5	TG1, pKT25-MshG Kan^{R}	SAD2272	This study
T18-MshG	Fig. S5	TG1, pUT18C-MshG Carb^{R}	SAD2273	This study
T25-Zip	Fig. S5	TG1, pKT25-leucine zipper Kan^{R}	SAD2238	[3]
T18-Zip	Fig. S5	TG1, pUT18C-leucine zipper Carb^{R}	SAD2241	[3]
P$_{tac}$-ribo-*mshA*^{parent} Δ*pilT*	Fig. S7A, S7B, S7C	E7946 Sm^{R}, ΔVC1807::Spec^{R}, *mshA*^{T70C}, Δ*lacZ*::P$_{tac}$-riboswitch-*mshA*^{T70C} Kan^{R}, Δ*pilT*::Tm^{R}	HKQ493 (SAD3334)	This study
P$_{tac}$-ribo-*mshA*^{parent} Δ*mshA*	Fig. S7C	E7946 Sm^{R}, ΔVC1807::Spec^{R}, *mshA*^{T70C}, Δ*lacZ*::P$_{tac}$-riboswitch-*mshA*^{T70C} Kan^{R}, Δ*mshA*::miniFRT	HKQ113 (SAD3336)	This study
P$_{tac}$-ribo-*mshA*^{P31S} Δ*mshA*	Fig. S7C	E7946 Sm^{R}, ΔVC1807::Spec^{R}, *mshA*^{T70C}, Δ*lacZ*::P$_{tac}$-riboswitch-*mshA*$^{T70C, P31S}$ Kan^{R}, Δ*mshA*::miniFRT	HKQ112 (SAD3337)	This study
mshA^{T70C} P$_{tac}$-ribo-*mshA*^{R32L} Δ*pilT*	Fig. S8	E7946 Sm^{R}, ΔVC1807::Spec^{R}, *mshA*^{T70C}, Δ*lacZ*::P$_{tac}$-riboswitch-*mshA*^{R32L} Kan^{R}, Δ*pilT*::Tm^{R}	TND3689 (SAD3420)	This study
mshA^{WT} P$_{tac}$-ribo-*mshA*^{R32L,T70C} Δ*pilT*	Fig. S8	E7946 Sm^{R}, Δ*lacZ*::P$_{tac}$-riboswitch-*mshA*$^{R32L, T70C}$ Kan^{R}, Δ*pilT*::Tm^{R}	TND3687 (SAD3421)	This study
mshA^{T70C} P$_{tac}$-ribo-*mshA*^{R32L,T70C} Δ*pilT*	Fig. S8	E7946 Sm^{R}, ΔVC1807::Spec^{R}, *mshA*^{T70C}, Δ*lacZ*::P$_{tac}$-riboswitch-*mshA*$^{R32L, T70C}$ Kan^{R}, Δ*pilT*::Tm^{R}	TND3688 (SAD3422)	This study
mshA^{T70C} P$_{tac}$-ribo-*mshA*^{V27F} Δ*pilT*	Fig. S8	E7946 Sm^{R}, ΔVC1807::Spec^{R}, *mshA*^{T70C}, Δ*lacZ*::P$_{tac}$-riboswitch-*mshA*^{V27F} Kan^{R}, Δ*pilT*::Tm^{R}	TND3683 (SAD3423)	This study
mshA^{WT} P$_{tac}$-ribo-*mshA*$^{V27F, T70C}$ Δ*pilT*	Fig. S8	E7946 Sm^{R}, Δ*lacZ*::P$_{tac}$-riboswitch-*mshA*$^{V27F, T70C}$ Kan^{R}, Δ*pilT*::Tm^{R}	TND3681 (SAD3424)	This study
mshA^{T70C} P$_{tac}$-ribo-*mshA*$^{V27F, T70C}$ Δ*pilT*	Fig. S8	E7946 Sm^{R}, ΔVC1807::Spec^{R}, *mshA*^{T70C}, Δ*lacZ*::P$_{tac}$-riboswitch-*mshA*$^{V27F, T70C}$ Kan^{R}, Δ*pilT*::Tm^{R}	TND3682 (SAD3425)	This study

REFERENCES

1. Floyd KA, Lee CK, Xian W, Nametalla M, Valentine A, Crair B, et al. c-di-GMP modulates type IV MSHA pilus retraction and surface attachment in Vibrio cholerae. Nat Commun. 2020;11(1):1549. Epub 2020/03/28. doi: 10.1038/s41467-020-15331-8. PubMed PMID: 32214098; PubMed Central PMCID: PMCPMC7096442.
2. Hughes HQ, Floyd KA, Hossain S, Anantharaman S, Kysela DT, Zöldi M, et al. Nitric oxide stimulates type IV MSHA pilus retraction in Vibrio cholerae via activation of the phosphodiesterase CdpA. Proceedings of the National Academy of Sciences. 2022;119(7). doi: 10.1073/pnas.2108349119.
3. Chlebek JL, Hughes HQ, Ratkiewicz AS, Rayyan R, Wang JC, Herrin BE, et al. PilT and PilU are homohexameric ATPases that coordinate to retract type IVa pili. PLoS Genet. 2019;15(10):e1008448. Epub 2019/10/19. doi: 10.1371/journal.pgen.1008448. PubMed PMID: 31626631; PubMed Central PMCID: PMCPMC6821130.
4. Heidelberg JF, Eisen JA, Nelson WC, Clayton RA, Gwinn ML, Dodson RJ, et al. DNA sequence of both chromosomes of the cholera pathogen Vibrio cholerae. Nature. 2000;406(6795):477-83. doi: 10.1038/35020000. PubMed PMID: 10952301.
5. Chlebek JL, Denise R, Craig L, Dalia AB. Motor-independent retraction of type IV pili is governed by an inherent property of the pilus filament. Proc Natl Acad Sci U S A. 2021;118(47). Epub 2021/11/19. doi: 10.1073/pnas.2102780118. PubMed PMID: 34789573; PubMed Central PMCID: PMCPMC8617508.
