# Supplementary material for: The PilT retraction ATPase promotes both extension and retraction of the MSHA type IVa pilus in Vibrio cholerae
Source: PLoS Genet. 2022 Dec 21;18(12):e1010561. doi: 10.1371/journal.pgen.1010561 (PMC9815625; doi:10.1371/journal.pgen.1010561)
Supplement: S2 Table — (PDF) [file pgen.1010561.s010.pdf]

## Table S2. Primers used in this study

| Primer name | Sequence (5' → 3') | Description |
|---|---|---|
| BBC1450 | CACTCAACGAGCTCAATACG | *mshA* F1 |
| BBC1453 | ATAGCCTTGCTGTTCATTTTGG | *mshA* R2 |
| ABD344 | GATTAGCAACGATTCTAGCGCAGGAG | ΔVC1807 F1 |
| ABD340 | gtcgacggatccccggaatACGTTTCATTAGTCACCTCTATTGT TAACTTGTTC | ΔVC1807 R1 |
| ABD341 | gaagcagctccagcctacaTAGTCGAAAATAAAAAAAAGAGG CTCGCCTC | ΔVC1807 F2 |
| ABD345 | CTTGCTAACCGTTGGTGTTACCAGC | ΔVC1807 R2 |
| DOG0400 | ACTTCTGGCTGAAGGTCAATTTTC | Δ*pilT/U* F1 |
| DOG0401 | gtcgacggatccccggaatCATTTAAATTCCTTAATAAAGTCTGGC | Δ*pilT* R1 |
| DOG0402 | gaagcagctccagcctacaTAGGTAGGTAAAGACAGATGGAG | Δ*pilT* F2 |
| DOG0403 | TCACGTGTTCGGCCAAAATC | Δ*pilT/U* R2 |
| ABD332 | GGCTGAACGTGGTTGTCGAAAATGAC | Δ*lacZ* F1 |
| ABD331 | GAACATGGGGTGTACGGCAGTGCtATTTAACGATGTGCGGGT TTTGCCAATCTTG | Δ*lacZ*::*lacIq* R1 |
| ABD255 | gaagcagctccagcctacaCCACAATAAGCCAGAGAGCCTTAAG | Δ*lacZ*::*lacIq* F2 |
| ABD256 | CCCAAATACGGCAACTTGGCG | Δ*lacZ* R2 |
| ABD841 | GGCACTGCCGTACACCCCATGTTCCGGTACCCGACACCATC GAATG | Amplify *lacIq* F |
| ABD842 | GGCTCTCTGGCTTATTGTGGGGGGAAACCTGTCGTGCCAGC TGC | Amplify *lacIq* R |
| BBC374 | TGGCAAAAAGCGAGAGAAGAAG | Δ*luxO* F1 |
| BBC375 | gtcgacggatccccggaatCATGAGGACATATTTTGTTTTCTGC | Δ*luxO* R1 |
| BBC376 | gaagcagctccagcctacaTAAGCGATGAGAGAATGGATCAAC | Δ*luxO* F2 |
| BBC377 | TCACACCCGAATTTCCATCATGC | Δ*luxO* R2 |
| pilA S81C F | AAAACTAATATTGAAGATTATATTGCGACAGAAGGC TgTTTTCCTGCAACAACTGCAGG | *pilA*$^{S56C}$ F |
| pilA R2 | CATTAATCGCGGTTTCAAAGTGCA | *pilA*$^{S56C}$ R2 |
| ABD648 | AACTAAGCTGCTGCAGATTGTC | *mshE* F1 |
| DOG0409 | gtcgacggatccccggaatTTTATTAATTGGCACTTTGTGTCTC | Δ*mshE* R1 |
| BBC742 | attccggggatccgtcgacCTGCAGTTCagaagcagctccagcctaca | miniFRT F |
| BBC743 | tgtaggctggagctgcttctGAACTGCAGgtcgacggatccccggaat | miniFRT R |
| BBC1748 | gaagcagctccagcctacaGAACCGATTTATCTGTAGGAGTAG | Δ*mshE* F2 |
| DOG0420 | TGTGTAGTCTAACGTTTCAATGC | *mshE* R2 |
| BBC1723 | caatttcacacaggatcccgggAGGAGGTATTTAAATGGATATCGCTGA GTTAC | ΔVCA0692::P$_{BAD}$-riboswitch-*pilT* insert F |
| BBC1724 | tgtaggctggagctgcttcCTAAAATGCTTTTAAATCCAACTCG | ΔVCA0692::P$_{BAD}$-riboswitch-*pilT* insert R |
| DOG0405 | gtcgacggatccccggaatCATCTGTCTTTACCTACCTAAAATGC | Δ*pilU* R1 |
| DOG0406 | gaagcagctccagcctacaTAGCGATGGTTACTTTGTGCG | Δ*pilU* F2 |
| HQP0001 | GGGCTCGGGTgctTCGACGACCTTGGCG | *pilT*$^{K136A}$ R1 |
| HQP0002 | CGCCAAGGTCGTCGAagcACCCGAGCCC | *pilT*$^{K136A}$ F2 |
| BBC2369 | GGCTGCCATAGTGGTCGAggcACCCGAACCGGTT GCGC | *pilU*$^{K134A}$ R1 |
| BBC2370 | GCGCAACCGGTTCGGGTgccTCGACCACTATGGCAGCC | *pilU*$^{K134A}$ F2 |
| BBC2288 | CGCAATGCCGAGCGCAATG | *pilT*$^{L201C}$ (*pilT* $^{slow}$) R1 |
| BBC2287 | CATTGCGCTCGGCATTGCGTGAAGACCCAGATG TGATCtgtGTCGGTGAGCTGCGTGAC | *pilT*$^{L201C}$ (*pilT* $^{slow}$) F2 |

| | | |
|---|---|---|
| HQP0141 | GTCACGCATCTCACCGACacAAATAATATCTGGGTCTT | *mshE*$^{L390C}$ (*mshE* $^{slow}$) R1 |
| HQP0142 | AAGACCCAGATATTATTTgtGTCGGTGAGATGCGTGAC | *mshE*$^{L390C}$ (*mshE* $^{slow}$) F2 |
| HQP0009 | GAAACGTGGCGCTGCTGTaatGGCCAAGATACCGAGAAT | *mshA*$^{V27I}$ R1 |
| HQP0008 | ATTCTCGGTATCTTGGCCattACAGCAGCGCCACGTTTC | *mshA*$^{V27I}$ F2 |
| HQP0013 | GAAACGTGGCGCTGCTGTaaaGGCCAAGATACCGAGAAT | *mshA*$^{V27F}$ R1 |
| HQP0012 | ATTCTCGGTATCTTGGCCtttACAGCAGCGCCACGTTTC | *mshA*$^{V27F}$ F2 |
| HQP0016 | TAAGAAACGTGGCGCcacTGTGACGGCCAAGAT | *mshA*$^{A29V}$ R1 |
| HQP0015 | ATCTTGGCCGTCACAgtgGCGCCACGTTTCTTA | *mshA*$^{A29V}$ F2 |
| HQP0019 | TTGCAGGTTTAAGAAACGagaCGCTGCTGTGACGGCCAA | *mshA*$^{P31S}$ R1 |
| HQP0018 | TTGGCCGTCACAGCAGCGtctCGTTTCTTAAACCTGCAA | *mshA*$^{P31S}$ F2 |
| HQP0022 | GCCTTGCAGGTTTAAGAAcagTGGCGCTGCTGTGACGGC | *mshA*$^{R32L}$ R1 |
| HQP0021 | GCCGTCACAGCAGCGCCActgTTCTTAAACCTGCAAGGC | *mshA*$^{R32L}$ F2 |
| HQP0025 | AGACGATTGCAACGCAATtgaCTCACTCGAAGTATCTAG | *mshA*$^{F119S}$ R1 |
| HQP0024 | CTAGATACTTCGAGTGAGtcaATTGCGTTGCAATCGTCT | *mshA*$^{F119S}$ F2 |
| BBC753 | atttcacacaggatcccgggAGGAGGTaacgtaATGGAATCTTATCTAAG TAAAAAAGTC | Δ*lacZ*::P$_{BAD}$-*dcpA* insert F |
| BBC754 | tgtaggctggagctgcttcTTAAGTCTCACTCATACCTGACAG | Δ*lacZ*::P$_{BAD}$-*dcpA* insert R |
| BBC1721 | caatttcacacaggatcccgggAGGAGGTCACAAAaTGCCAATTAATAA ACTGC | Δ*lacZ*::P$_{BAD}$-*mshE** insert F |
| BBC4463 | aattcgcattaaggaggtaccGTGCCAATTAATAAACTGCGTAAAC | Δ*lacZ*::pLTetO-*mshE** insert / MIDDLE2 F |
| BBC1722 | tgtaggctggagctgcttcCTACAGATAAATCGGTTCAACC | Δ*lacZ*::P$_{BAD}$-*mshE** R insert or Δ*lacZ*::pLTetO-*mshE** insert / MIDDLE2 R |
| ABD332 | GGCTGAACGTGGTTGTCGAAAATGAC | Δ*lacZ*::pLTetO-*mshE** UP F1 |
| BBC2511 | CTCATTAGGCACCCCAGGC | Δ*lacZ*::pLTetO-*mshE** UP R1 |
| BBC2917 | gcctggggtgcctaatgagAGACGCGGCTAGCCAAATC | Δ*lacZ*::pLTetO-*mshE** MIDDLE1 F |
| BBC2919 | GGTACCTCCTTAATGCGAATT | Δ*lacZ*::pLTetO-*mshE** MIDDLE1 R |
| ABD255 | gaagcagctccagcctacaCCACAATAAGCCAGAGAGCCTTAAG | Δ*lacZ*::pLTetO-*mshE** DOWN F2 |
| ABD256 | CCCAAATACGGCAACTTGGCG | Δ*lacZ*::pLTetO-*mshE** DOWN R2 |
| BBC2022 | TGCCGATCTCGTTTATGGACG | Δ*vps-rbmA* locus F1 |
| BBC2023 | gtcgacggatccccggaatTGCCATTTTGATTGCCTCTGG | Δ*vps-rbmA* locus R1 |
| BBC2024 | gaagcagctccagcctacaCAAAGAGAGCCTTATTAGGCTCTC | Δ*vps-rbmA* locus F2 |
| BBC2025 | TGAAAGAGGTTGCTCTAGAACTCG | Δ*vps-rbmA* locus R2 |
| BBC2975 | caatttcacacaggatcccgggaggaggtGGTACCATGAGTAAAAAAATC AAACTACC | ΔVCA0692::P$_{BAD}$-*mf-lon* insert F |
| BBC2918 | tgtaggctggagctgcttcTCCTACTCAGGAGAGCGTTCAC | ΔVCA0692::P$_{BAD}$-*mf-lon* insert R |
| BBC4364 | ACTCTCGGTAAACTTGCACG | *mshJ* F1 |
| BBC4365 | tccaccacttccacctgcACCACCGATAAACTCCTCTC | *mshJ*-X R1 |
| BBC4366 | gcaggtggagcaggtggaGGCAGTCGAGAGGAGTTTATC | *mshJ*-X F2 |

| | | |
|---|---|---|
| BBC2950 | TCATGGTTTGCTCCATCCC | *mshJ* R2 |
| BBC1835 | gcaggtggaagtggtggaGTGAGCAAGGGCGAGGAGG | *mCherry* F |
| BBC1836 | gattgcagcTTACTTGTACAGCTCGTCC | *mCherry* R |
| BBC2975 | caatttcacacaggatcccgggaggaggtGGTACCATGAGTAAAAAAATC AAACTAC | *mf-lon* insert F |
| BBC2918 | tgtaggctggagctgcttcTCCTACTCAGGAGAGCGTTCAC | *mf-lon* insert R |
| BBC2927 | cttcgtttttgttcgccgcAAATGCTTTTAAATCCAACTCGATT | *pilT-pdt2* R1 |
| BBC2932 | gcggcgaacaaaaacgaagaaaacaccaacgaagtgccgacctttatgctgaacgcg ggccaggcgaactacctgtctcaataagaagcagctccagcctaca | *pdt2* F |
| BBC2933 | tgtaggctggagctgcttcttattgagacaggtagttcgcctggcccgcgttcagcataaag gtcggcacttcgttggtgttttcttcgtttttgttcgccgc | *pdt2* R |
| DOG0402 | gaagcagctccagcctacaTAGGTAGGTAAAGACAGATGGAG | *pilT-pdt2* F2 |
| BBC2513 | gaagcagctccagcctacaGATCCCCGGGTACCTAAGTAAC | BACTH pKT25-X F |
| BBC2512 | tccacctgctccacctgcCTCTAGAGTCGACCCTGCAG | BACTH pKT25-X R |
| BBC2515 | gaagcagctccagcctacaCCGAGCTCGAATTCATCGATATAAC | BACTH pUT18C-X F |
| BBC2514 | tccacctgctccacctgcCTCTAGAGTCGACCTGCAG | BACTH pUT18C-X R |
| BBC2517 | gcaggtggaagtggtggaGATCCCCGGGTACCGAGC | BACTH X-pKT25/pUT18C F |
| BBC2516 | tccacctgctccacctgcCTCTAGAGTCGACCTGCAGG | BACTH X-pKT25/pUT18C R |
| JCP027 | gcaggtggagcaggtggaGATATCGCTGAGTTACTGGAATTTAG | BACTH TXX-PilT F |
| BBC1724 | tgtaggctggagctgcttcCTAAAATGCTTTTAAATCCAACTCG | BACTH TXX-PilT R |
| BBC2434 | gcaggtggagcaggtggaGAGTTGAATCAATATCTGGATGG | BACTH TXX-PilU F |
| BBC1726 | tgtaggctggagctgcttcCTACTCCATATCAATCTTCACATTC | BACTH TXX-PilU R |
| HQP0355 | gcaggtggagcaggtggaCCAATTAATAAACTGCGTAAACG | BACTH MshE-TXX F |
| BBC2316 | tccaccacttccacctgcCAGATAAATCGGTTCAACCAAACC | BACTH MshE-TXX R |
| BBC2638 | gcaggtggagcaggtggaCTCACCAACCTTGTTGCTATC | BACTH TXX-PilB F |
| BBC2639 | tgtaggctggagctgcttcTTAAAAGTAGAGCACACGCTG | BACTH TXX-PilB R |
| BBC2526 | gcaggtggagcaggtggaGCGACGTTTTATTATCAGGG | BACTH TXX-MshG F |
| BBC2532 | tgtaggctggagctgcttcTTAGCCCTTGAGTGCATCCATC | BACTH TXX-MshG R |
| BBC2617 | cctgctaaggaggtaacaacaagATGGTAATAATGAAAAGACAAGGTG | Δ*lacZ*::$P_{tac}$-riboswitch-*mshA* insert F |
| BBC1712 | tgtaggctggagctgcttcTTATTGCGCTGGTTTACCAC | Δ*lacZ*::$P_{tac}$-riboswitch-*mshA* insert R |
| ABD653 | gtcgacggatccccggaatCATCTCTCTTTCATGTGAATACGCA GC | Δ*mshA* R1 |
| ABD654 | gaagcagctccagcctacaGCGCAATAATTTAAATATGGCTCG TGC | Δ*mshA* F2 |
| HQP0129 | GTATATGCTGTGTAGTCTAAcgtTTCAATGCCTTTAATTGCAG | *mshA*$^{C70T}$ R1 |
| HQP0130 | CTGCAATTAAAGGCATTGAAacgTTAGACTACACAGCATATAC | *mshA*$^{C70T}$ F2 |
